# Supporting mental health during pregnancy: The feasibility of a combined peer support and mindfulness intervention, a quasi-experimental pre-post feasibility study

**DOI:** 10.18332/ejm/226733

**Published:** 2026-09-02

**Authors:** Sudjit Liblub, Karen McLaughlin, Kirsty Pringle, Allison Cummins

**Affiliations:** 1 Ramathibodi School of Nursing, Faculty of Medicine Ramathibodi Hospital, Mahidol University, Bangkok, Thailand; 2 School of Nursing and Midwifery, College of Health Medicine and Wellbeing, The University of Newcastle, Callaghan, Australia; 3 School of Nursing and Midwifery, Western Sydney University, WSU Clinical School, Blacktown Hospital, Sydney, Australia; 4 School of Biomedical Sciences and Pharmacy, College of Health Medicine and Wellbeing, The University of Newcastle, Callaghan, Australia; 5 Mothers and Babies Research Program, Hunter Medical Research Institute, New Lambton Heights, Australia

**Keywords:** anxiety, depression, mindfulness, peer group, pregnancy

## Abstract

**INTRODUCTION:**

Pregnancy is a critical period of heightened vulnerability to mental health challenges. Peer support and mindfulness have individually shown promise as non-pharmacological perinatal mental health strategies, yet their combined potential remains underexplored. This study evaluated the feasibility of a novel intervention integrating peer support with a mindfulness application.

**METHODS:**

A quasi-experimental pre–post feasibility design was conducted between April 2024 and January 2025 at an antenatal clinic in a public hospital in New South Wales, Australia, using convenience sampling across two sequential recruitment phases. The 4-week intervention combined mutual peer support sessions with guided mindfulness practice via the Headspace app, enrolling pregnant women at elevated risk of anxiety and depression (priority group) and those from the general pregnant population. Validated self-report measures (DASS-21, FFMQ-15, MSPSS) and salivary cortisol samples were collected pre- and post-intervention. Wilcoxon signed-rank and Mann-Whitney U tests examined within- and between-group changes; open-ended post-intervention survey responses were analyzed using content analysis.

**RESULTS:**

Thirteen pregnant women participated, achieving a 100% retention rate despite recruitment challenges. A statistically significant reduction in stress was observed in the general group compared to the priority group (median [IQR]: 22 [14–30] vs 8 [2–20]; p<0.050). No significant pre-to-post changes were found in anxiety, depression, mindfulness, or perceived social support in either group (p>0.05). Qualitative findings highlighted that the intervention fostered emotional support, connectedness, and mindfulness engagement, with participants expressing willingness to recommend the program.

**CONCLUSIONS:**

Combining peer support with a mindfulness application appears feasible and acceptable for pregnant women and warrants evaluation in adequately powered randomized controlled trials.

## INTRODUCTION

The perinatal period, spanning pregnancy to one year postpartum, is a vulnerable time marked by substantial social, psychological, and physiological stress^1^. Approximately 12% of perinatal women experience depression, and 13.7% report anxiety during pregnancy, with prevalence peaking in the first six months postpartum, particularly in high-income countries^2^. In Australia, depression affects one in ten pregnant women, a burden that has remained persistently high despite growing policy attention and increased screening activity, reflecting ongoing gaps in accessible mental healthcare within the perinatal period^1^. Anxiety, depression, and stress during pregnancy have both immediate and long-term consequences, including increased risk of preterm birth^3^ and greater healthcare utilization, contributing to national healthcare costs^1^. Longer term, perinatal mental health difficulties can disrupt maternal–infant bonding and negatively affect children’s emotional and overall development^4^. Antenatal and postnatal anxiety are significantly associated with internalizing and externalizing problems in children, underscoring the intergenerational burden of perinatal mental illness and the importance of early, effective intervention^1,3^.

Current approaches to supporting perinatal mental health are anchored in clinical guidelines that recommend universal screening followed by stepped psychological and pharmacological interventions for those identified as at risk^1^. However, pharmacological options are frequently avoided by pregnant and breastfeeding women due to concerns about fetal and infant safety, and many women express a strong preference for non-pharmacological approaches to managing perinatal distress^5^. In recent years, non-pharmacological approaches to supporting maternal mental health and wellbeing have become increasingly popular. Peer support is a potentially effective non-pharmacological prevention for perinatal depression^6^. Mindfulness-based interventions (MBIs) have also been recognized for their positive impact on mental health in pregnant women, functioning as psychological interventions^5^. Mindfulness training through smartphone applications has shown evidence of improving perinatal depression, particularly among those at risk^7^. Peer support specifically addresses social isolation and provides emotional validation, while mindfulness targets individual coping through regulation of attention and present-moment awareness^6,7^. Combining these approaches may offer synergistic and complementary benefits by targeting both social connection and individual coping processes^8^. Moreover, high attrition rates in standalone mindfulness programs may be mitigated by the added social accountability of peer support groups^9^. However, to date, no research has examined whether combining peer support with mindfulness applications can improve mental health outcomes during pregnancy. Furthermore, no single study has evaluated both the psychological and physiological effects of such a combined intervention in perinatal women. Such research is needed because psychological stress during pregnancy is associated with adverse maternal and fetal outcomes, including increased anxiety and depression and altered physiological stress regulation^10^.

Cortisol is a well-established and accessible indicator of physiological stress because it reflects activation of the hypothalamic–pituitary–adrenal (HPA) axis^10,11^. Therefore, salivary cortisol was included in the present study as an objective biomarker alongside validated psychometric measures. Combining objective biomarkers with validated self-report measures provides a more comprehensive assessment of intervention effects, as self-report tools alone may not fully capture the biological stress response^12^.

To our knowledge, no previous study has examined the feasibility of combining peer support with a smartphone-based mindfulness application in pregnant women. This quasi-experimental pre-post feasibility study aimed to evaluate: 1) recruitment and retention rates; 2) participant feedback on the intervention; and 3) preliminary changes in psychological outcomes (anxiety, depression, stress), mindfulness, perceived social support, and salivary cortisol.

## METHODS

### Study design and setting

This study used a non-randomized quasi-experimental pre-post design to evaluate the feasibility of a novel peer support intervention combined with a mindfulness application. The study was conducted at an antenatal clinic within a public hospital in New South Wales, Australia. As this was a feasibility study, no formal *a priori* sample size calculation was performed. The final sample size was determined by recruitment feasibility over the study period, consistent with the recommendation that a minimum of 12 participants represents a practical benchmark for pilot and feasibility research to estimate intervention parameters and inform future trial design^13^. The findings are intended to inform the design of future fully powered randomized controlled trials rather than to detect efficacy.

### Participants and recruitments

A convenience sampling strategy was adopted, recruiting eligible pregnant women from the antenatal clinic across two sequential phases with different inclusion criteria, as described below.

Initial inclusion criteria targeted women identified as having a higher chance of experiencing anxiety and/or depression, defined as an EPDS score ≥10 or self-reported history of anxiety and/or depression at recruitment (Priority Population). Due to unexpectedly low enrolment despite multiple recruitment strategies, ethical approval was amended to expand recruitment to all pregnant women attending the antenatal clinic who did not screen positive on the Edinburgh Postnatal Depression Scale (EPDS <10) and did not self-report a prior history of anxiety or depression (General Population). This decision was made to assess the feasibility and acceptability of the intervention across a broader antenatal population and to generate sufficient data to inform future research design. Across both phases, participants were required to be less than 30 weeks’ gestation, aged ≥18 years, and able to read and understand English. Exclusion criteria included multiple pregnancy, pregnancy complications, requirement for specialist care, or complex mental health conditions, defined as current specialist mental health service involvement, psychotic disorder, active suicidal ideation under clinical management, or current inpatient psychiatric treatment.

Pregnant women were recruited from a public hospital in New South Wales, Australia, with two data collection points over a 10-month period. The first recruitment phase (April–September 2024) involved midwives identifying eligible participants during routine antenatal booking appointments using the EPDS; women scoring ≥10 or who reported a prior history of clinically diagnosed and/or treated anxiety or depression during the antenatal history-taking at the booking, were referred to the research team who provided study information and obtained written informed consent. Recruitment was also supported through posters in the antenatal clinic waiting room and social media (e.g. maternal Facebook groups) to reach women who self-reported a history of anxiety and/or depression.

Due to low enrolment, ethical approval was amended to include all pregnant women. During the second recruitment phase (October 2024–January 2025), the student researcher approached women in the clinic waiting room to introduce the study, with additional promotion via social media, posters, and flyers included in booking-in packs distributed by midwives.

### Peer support group combined with a mindfulness application intervention

The intervention combined peer support groups with the Headspace mindfulness application, an evidence-based mental health tool providing practical guidance for integrating mindfulness into daily life during pregnancy. Peer groups aimed to foster mutual support while practicing mindfulness together, with a focus on coping strategies during pregnancy. Sessions followed a structured format developed from previous qualitative data findings collected via interviews with pregnant women who had shared similar experiences to inform the sessions (first phase of the study). The intervention was designed to encourage engagement through shared experiences, positive emotions, and idea exchange (Tables 1 and 2).

**Table 1:** Overview of structure of the peer support group combined with a mindfulness app

*Time (min)*	*Activities*	*Details*
5	Welcome and introduction	Each session began with an acknowledgment of Country and a welcome to the group. The concept of a support group was introduced.
15	Warm-up activity (varies each week)	A brief round of introductions was conducted, giving each participant the opportunity to share their name and due date. The following are examples of activities that were used: Ice-Breaker: Using the *Instant Delight* tool (NSW Ministry of Health, 2024), participants responded to questions such as, ‘What are things you value, that touch your heart, that brighten your day?’ and ‘What’s one thing that always brought a smile to your face, no matter how tough your day had been?’. Emotional check-in: Using *Visual Inquiry* (Dewar et al. 2020), participants selected an image that helped them express how they had been feeling about their pregnancy experience. Emotional Words Activity (NSW Ministry of Health, 2024): Participants were invited to choose two emotional words that summed up their pregnancy experience so far – one positive and one negative. Reflection – *‘In the Know’* Tool (NSW Ministry of Health, 2024): To recap what they had learned from the group, participants reflected on one thing they were proud or excited to know, one thing they were getting to know, and one thing they did not know yet. These activities helped participants get to know one another and feel more comfortable sharing their personal stories.
20	Topic discussion and peer-to-peer support	Members were invited to share their experiences and to offer support and advice to one another. The facilitator shared topic questions with the group to encourage discussion and promote mutual peer support. The questions were relevant and aligned with the session topic, as shown in Table 2.
10	Practice mindfulness meditation	The facilitator led the group in a mindfulness activity. The mindfulness exercise used the pregnancy pack from the Headspace app. The Headspace courses for pregnancy taught pregnant women how to apply mindfulness in their daily lives, using techniques such as breath awareness, body scanning, connection with the baby, reassurance in mind, and visualization (e.g. visualizing images such as the sun shining on the entire body).
10	Closing the meeting	The group reviewed any action items or goals that were set during the meeting, and the facilitator provided information about the next meeting.

**Table 2:** Overview of topic discussions for the peer support group combined with a mindfulness app

*Week*	*Topic*	*Example of discussion topics*
1	Introduction to the intervention and focusing on one’s personal health and well-being	Outlined peer support combined with a mindfulness app; established ground rules to promote trust and confidentiality among participants; and encouraged participants to share things that had helped them feel relaxed, what they usually did to distract themselves from worries, and activities or things that had motivated them to get up and move.
2	Ideas related to physical and emotional common discomforts of pregnancy	Shared physical discomforts or challenges they had experienced during pregnancy and how they had coped with them; and shared specific activities or hobbies they had used to distract themselves from pregnancy-related pain or discomfort.
3	Increasing helpful thoughts that affect my baby and myself	Shared one personal strength they used to describe themselves; one quality they hoped their child would inherit from them or their partner; and one skill they wished to learn or develop in order to teach their baby.
4	Transition to motherhood and community and mindfulness-based resources	Shared how their support network (family, friends, etc.) had been helpful during their transition to motherhood; described individuals who had played a significant role in supporting them through the journey; and shared resources, books, or online communities they had found valuable during pregnancy.

Each weekly session comprised: 1) a 5-minute welcome; 2) a 15-minute warm-up activity; 3) a 20-minute topic discussion and peer-to-peer support segment; 4) a 10-minute guided mindfulness meditation using the Headspace pregnancy pack; and 5) a 10-minute closing review.

The intervention was delivered over four weeks, comprising weekly one-hour group sessions facilitated by the researchers. The first session was held in person at the hospital, with subsequent sessions conducted either in person or online via the University’s secure Zoom platform, according to participant preference.

Participants also accessed the Headspace application (iOS, Android, or web) free of charge via a voucher funded by Headspace Inc.

### Instruments

*Psychological outcome measures*

Psychological outcomes were assessed using the 21-item Depression Anxiety Stress Scale (DASS-21), which has demonstrated acceptable reliability in Australian pregnant women (α=0.62–0.75)^14^. Mindfulness was measured using the Five Facet Mindfulness Questionnaire–15 (FFMQ-15), comprising observing, describing, acting with awareness, and non-judging subscales, with internal consistency ranging from α=0.75 to 0.91^15^. Perceived social support was assessed using the Multidimensional Scale of Perceived Social Support (MSPSS), a 12-item scale with excellent reliability (α=0.933)^16^.

*Biological measure*

To examine psychobiological parameters, salivary cortisol was used as an objective physiological indicator of stress, reflecting hypothalamic–pituitary–adrenal (HPA) axis activation and biological stress responses^11^.

*Feasibility and acceptability measures*

Feasibility and acceptability of the intervention were evaluated using two post-intervention surveys: 1) Likert-type questions assessing overall satisfaction, based on previous evaluations of a mindfulness application^17^, and the peer support group^18^; and 2) Open-ended questions were used to elicit participants’ reflections on their experiences of the intervention, with a focus on perceived value, while also allowing participants to comment on any aspect of their experience, including suggestions for improvement.

### Data collection

Quantitative data were collected at baseline (pre-test) and post-intervention (post-test). Participants completed a demographic questionnaire and validated measures (DASS-21, FFMQ-15, MSPSS) before and after the four-week intervention, followed by a brief post-intervention evaluation survey. Qualitative data were obtained through open-ended questions designed using a strengths-based, appreciative inquiry–informed approach to explore participants’ experiences and perceptions of the intervention. Participants were asked: ‘What’s the most valuable and useful aspect of the peer support group combined with using a mindfulness application?’ and ‘Anything else you’d like to share?’.

### Biological sample collection and salivary cortisol assays

Salivary cortisol was collected immediately before and after peer support–mindfulness sessions on the first and last intervention days. All sessions were held in the evening, with post-session samples collected at similar times to minimize diurnal variation. To minimize potential confounders, participants were asked to refrain from eating, drinking (except water), and consuming caffeine for at least 60 minutes prior to saliva collection. Participants used the passive drool method, and samples were frozen at -80°C until analysis. Cortisol concentrations were measured using a cortisol enzyme immunoassay (ELISA) kit from Salimetrics (State College, PA), following the manufacturer’s instructions.

### Data analysis

Data were analyzed using SPSS version 29.0.2.0 (IBM, Armonk, NY). Descriptive statistics summarized demographic characteristics and post-intervention evaluation responses, with continuous data reported as mean with standard deviation (SD), and categorical data as frequencies and percentages. As normality was violated for psychological measures (DASS-21, FFMQ-15, MSPSS), nonparametric analyses were applied. Within-group pre–post differences (priority and general groups) were examined using Wilcoxon signed-rank tests, with outcomes reported as medians and interquartile ranges (IQRs). Between-group differences in change scores (post–pre) were assessed using the Mann–Whitney U test. Statistical significance was set at p<0.05. For salivary cortisol, data normality was assessed, and a two-way ANOVA examined the effects of session (first vs last) and group (priority vs general). Pearson correlation coefficients assessed associations between cortisol levels and the DASS-21 stress subscale.

There were no missing data for questionnaire outcomes, as all participants completed both pre- and post-intervention surveys. For salivary cortisol, two participants in the general group had incomplete data pairs and were excluded from cortisol analyses, resulting in 11 participants with complete cortisol data. No imputation was performed.

Open-ended comments were analyzed using content analysis to explore participants’ experiences, involving systematic coding of written data to identify categories and their frequency^19^. A deductive approach was applied, using social support theory as the analytical framework, enabling a nuanced examination of how participants perceived and benefited from the intervention^20^. Social support was conceptualized as comprising functional and structural components, with functional support including emotional support (expressions of empathy, care, and encouragement), instrumental support (tangible acts of aid or service), informational support (provision of advice, suggestions, and shared knowledge), and appraisal support (constructive feedback to guide progress toward specific goals or tasks)^21^. Together, these elements may produce a ‘buffering effect’, enhancing health outcomes and reducing stress^21^, and guiding interpretation of participants’ experiences within the intervention.

## RESULTS

### Quantitative findings

*Response, enrolment, uptake and retention rates*

**Figure 1:**
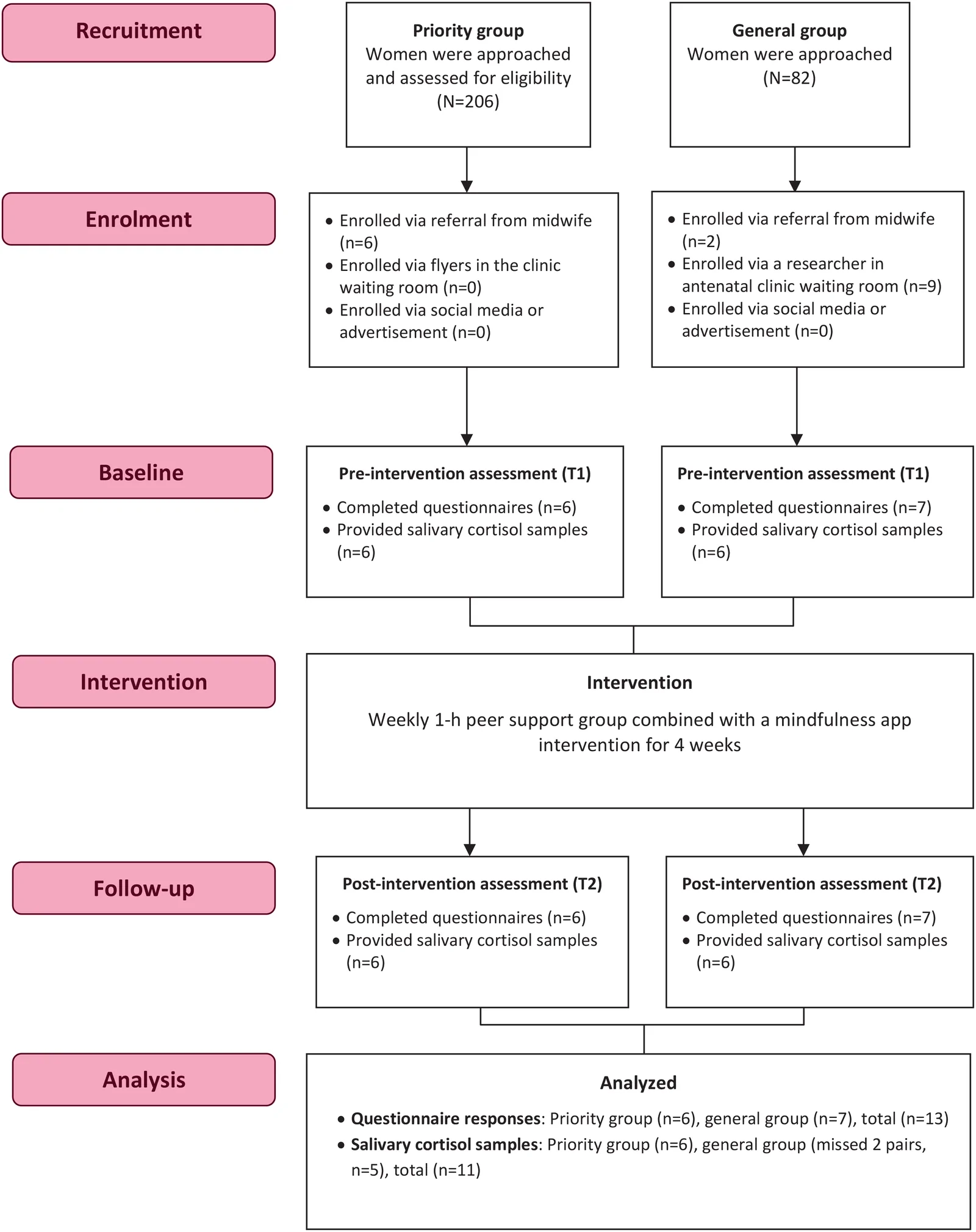
Flowchart of participant selection

A total of 13 pregnant women participated in this feasibility study (six in the priority group and seven in the general group) and were included in the analysis. Figure 1 presents the study flowchart and recruitment outcomes. Response rates were 2.9% in the priority group (6/206 approached) and 13.4% in the general group (11/82 approached). The enrolment rate in the priority group was 23.1% (6/26 eligible); the enrolment rate for the general group could not be calculated due to unknown eligibility numbers. Retention, defined as continued engagement without withdrawal, was 100%, with all 13 participants attending at least three of four sessions. All participants completed pre- and post-intervention questionnaires, and all data were included in the analysis. For cortisol sampling, all six priority group participants provided paired samples; in the general group, one participant missed the pre-test and another the post-test, resulting in five complete pairs. Overall, 11 of 13 participants (84.6%) provided complete salivary cortisol samples.

*Participant characteristics*

Participant characteristics are presented in Table 3. Ages ranged from 19 to 35 years (mean=34.23 years, SD=4.64), with a mean gestational age of 21.08 weeks (SD=3.82). Most participants were experiencing their first pregnancy (69.23%) and were born in Australia (61.54%). Almost all (92.31%) reported paid employment, and the majority (85%) had no prior experience using mindfulness applications.

**Table 3:** Characteristics of the participants, antenatal clinic, NSW, Australia, April 2024–January 2025 (N=13)

*Characteristics*	*n (%)*
**Age** (years), mean (SD)	34.23 (4.64)
**Gestational age** (weeks), mean (SD)	21.08 (3.82)
**Parity**	
No prior births	9 (69.23)
≥1 prior births	4 (30.77)
**Born in Australia**	
Yes	8 (61.54)
No	5 (38.46)
**English first language**	
Yes	9 (69.23)
No	4 (30.77)
**Employment status**	
Paid work	12 (92.31)
Not in paid work	1 (7.69)
**Current access to mindfulness app**	
Yes	2 (15)
No	11 (85)

*Within-group comparison of outcomes*

No statistically significant post-intervention changes were observed in DASS-21, mindfulness (FFMQ-15), or perceived social support (MSPSS) scores (p>0.05). Both groups showed small, non-significant numerical increases in mindfulness and perceived social support scores; a slightly larger numerical change was observed in the Friend subscale of the MSPSS, though this did not reach statistical significance (p>0.05) (Table 4).

**Table 4:** Within-group pre- and post- Depression Anxiety Stress Scales (DASS-21), Five Facet Mindfulness Questionnaire (FFMQ-15) and Multidimensional Scale of Perceived Social Support (MSPSS), antenatal clinic, NSW, Australia, April 2024–January 2025 (N=13)

*Subscale*	*Group*	*Pre intervention*	*Post intervention*	*Median change*	*Wilcoxon’s signed rank*	
		*Median (IQR)*	*Median (IQR)*		*Z*	*p*
**DASS-21**						
Depression	Priority	8.0 (0–32)	8.0 (4–30)	0	1.36	0.581
	General	4.0 (2–12)	2.0 (2–14)	-2	0.738	0.461
Anxiety	Priority	9.00 (4–18)	8.00 (2–14)	-1	0.736	0.461
	General	4.0 (2–12)	4.0 (2–14)	0	6.49	0.516
Stress	Priority	16.00 (10–26)	22.00 (14–30)	6	1.261	0.207
	General	10.00 (4–18)	8.0 (2–20)	-2	1.364	0.172
**FFMQ-15**	Priority	47.50 (42–58)	49.00 (36–53)	1.5	1.22	0.313
	General	49.00 (45–58)	53.00 (43–63)	4	1.26	0.250
Observing	Priority	10.50 (7–11)	10.00 (7–14)	-0.5	0.00	1.00
	General	8.00 (6–12)	11.00 (8–12)	3	1.78	0.094
Descripting	Priority	10.00 (7–14)	9.00 (8–14)	-1	0.31	0.500
	General	12.00 (9–13)	12.00 (7–15)	0	0.11	1.00
Acting with awareness	Priority	8.00 (6–13)	8.50 (4–14)	0	0.31	0.844
	General	9.00 (6–11)	9.00 (7–12)	0	0.84	0.500
Non-judging	Priority	9.50 (8–12)	10.00 (6–13)	0.5	0.41	0.813
	General	11.00 (8–13)	12.00 (5–15)	1	0.68	0.578
Non-reactivity	Priority	9.50 (9–10)	9.00 (8–12)	-0.5	0.00	1.000
	General	12.00 (9–13)	11.00 (8–15)	-1	0.26	0.859
**MSPSS**	Priority	5.79 (4.6–6.6)	5.87 (4.9–6.4)	0.08	-0.10	1.00
	General	5.87 (4.9–6.4)	6.25 (4.2–7.0)	0.38	-0.10	1.00
Special one	Priority	6.62 (6.0–7.0)	6.0 (3.5–7.0)	-0.62	-0.67	0.625
	General	6.62 (5.2–7.0)	7.0 (3.5–7.0)	0.38	-0.67	0.625
Family	Priority	5.75 (3.0–7.0)	6.0 (5.0–7.0)	0.25	-0.27	1.00
	General	6.0 (5.0–7.0)	5.75 (4.7–7.0)	-0.25	-0.27	1.00
Friends	Priority	5.25 (4.0–6.0)	5.5 (4.75–5.8)	0.25	-0.10	1.00
	General	5.25 (4.2–7.0)	6.00 (4.0–7.0)	0.75	-0.10	1.00

IQR: interquartile range. Priority group: pregnant women with EPDS score ≥10 or self–reported history of anxiety and/or depression. General group: all other pregnant women with EPDS score <10 and self–reported no history of anxiety and/or depression recruited from the antenatal clinic. Pre: baseline assessment conducted prior to Session 1. Post: assessment conducted following Session 4 (final session). Statistical significance threshold p<0.05.

*Between-group comparison of outcomes*

A statistically significant between-group difference was observed for DASS-21 scores post-intervention (p=0.004), with a large effect size (r=0.76). Pre-intervention stress scores showed a marginal, non-significant difference between groups (p=0.057). No significant between-group differences were found for depression, anxiety, mindfulness (FFMQ-15), or perceived social support (MSPSS) at any time point (all p>0.05) (Table 5).

**Table 5:** Comparing the changes in Depression Anxiety Stress Scales (DASS-21), Five Facet Mindfulness Questionnaire (FFMQ-15) and Multidimensional Scale of Perceived Social Support (MSPSS) scores between groups, antenatal clinic, NSW Australia, April 2024–January 2025 (N=13)

*Subscale*	*Time*	*Priority group*	*General group*	*Median difference*	*Mann-Whitney U*	*p*
		*Median (IQR)*	*Median (IQR)*			
**DASS–21**						
Depression	Pre	8.0 (0–32)	4.0 (2–12)	4	15.5	0.457
	Post	8.0 (4–30)	2.0 (2–14)	6	9.5	0.099
Anxiety	Pre	9.00 (4–18)	4.0 (2–12)	5	12.5	0.247
	Post	8.00 (2–14)	4.0 (2–14)	4	16.5	0.571
Stress	Pre	16.00 (10–26)	10.00 (4–18)	6	7.5	0.057
	Post	22.00 (14–30)	8.0 (2–20)	14	2.0	**0.004**
**Overall FFMQ-15**	Pre	47.5 (42–58)	49.0 (45–58)	1.5	16.0	0.515
	Post	49.0 (36–53)	53.0 (43–63)	4	11.5	0.186
Observing	Pre	10.5 (7–11)	8.0 (6–12)	2.5	11.0	0.177
	Post	10 (7–14)	11.0 (8–12)	1	17.0	0.604
Descripting	Pre	10.0 (7–14)	12.0 (9–13)	2	19.0	0.800
	Post	9.0 (8–14)	12.0 (7–15)	3	14,0	0.345
Acting with awareness	Pre	8.0 (6–13)	9.0 (6–11)	1	19.5	0.857
	Post	8.5 (4–14)	9.0 (7–12)	0.5	14.5	0.384
Non-judging	Pre	9.5 (8–12)	11 (8–13)	1.5	14.0	0.361
	Post	10.0 (6–13)	12.0 (5–15)	2	15.0	0.433
Non-reactivity	Pre	9.5 (9–10)	12.0 (9–13)	2.5	7.5	0.073
	Post	9.0 (8–12)	11.0 (8–15)	2	10.0	0.132
**Overall MSPSS**	Pre	5.79 (4.58–6.58)	5.41 (4.42–7.00)	0.38	20.0	0.945
	Post	5.87 (4.92–6.42)	6.25 (4.17–7.00)	0.38	11.5	0.193
Special one	Pre	6.62 (6.00–7.00)	6.00 (3.50–7.00)	0.62	16.0	0.525
	Post	6.62 (5.25–7.00)	7.00 (3.50–7.00)	0.38	15.0	0.404
Family	Pre	5.87 (2.25–7.00)	5.75 (4.75–7.00)	0.12	15.5	0.457
	Post	5.75 (3.00–7.00)	6.00 (5.00–7.00)	0.25	21.0	1.000
Friends	Pre	5.25 (4.00–6.00)	5.25 (4.25–7.00)	0.00	15.5	0.462
	Post	5.50 (4.75–5.75)	6.00 (4.00–7.00)	0.50	11.0	0.169

IQR: interquartile range. Priority group: pregnant women with EPDS score ≥10 or self-reported history of anxiety and/or depression. General group: all other pregnant women with EPDS score <10 and self-reported no history of anxiety and/or depression recruited from the antenatal clinic. Pre: baseline assessment conducted prior to Session 1. Post: assessment conducted following Session 4 (final session). Statistical significance threshold p<0.05.

*Cortisol levels*

**Figure 2:**
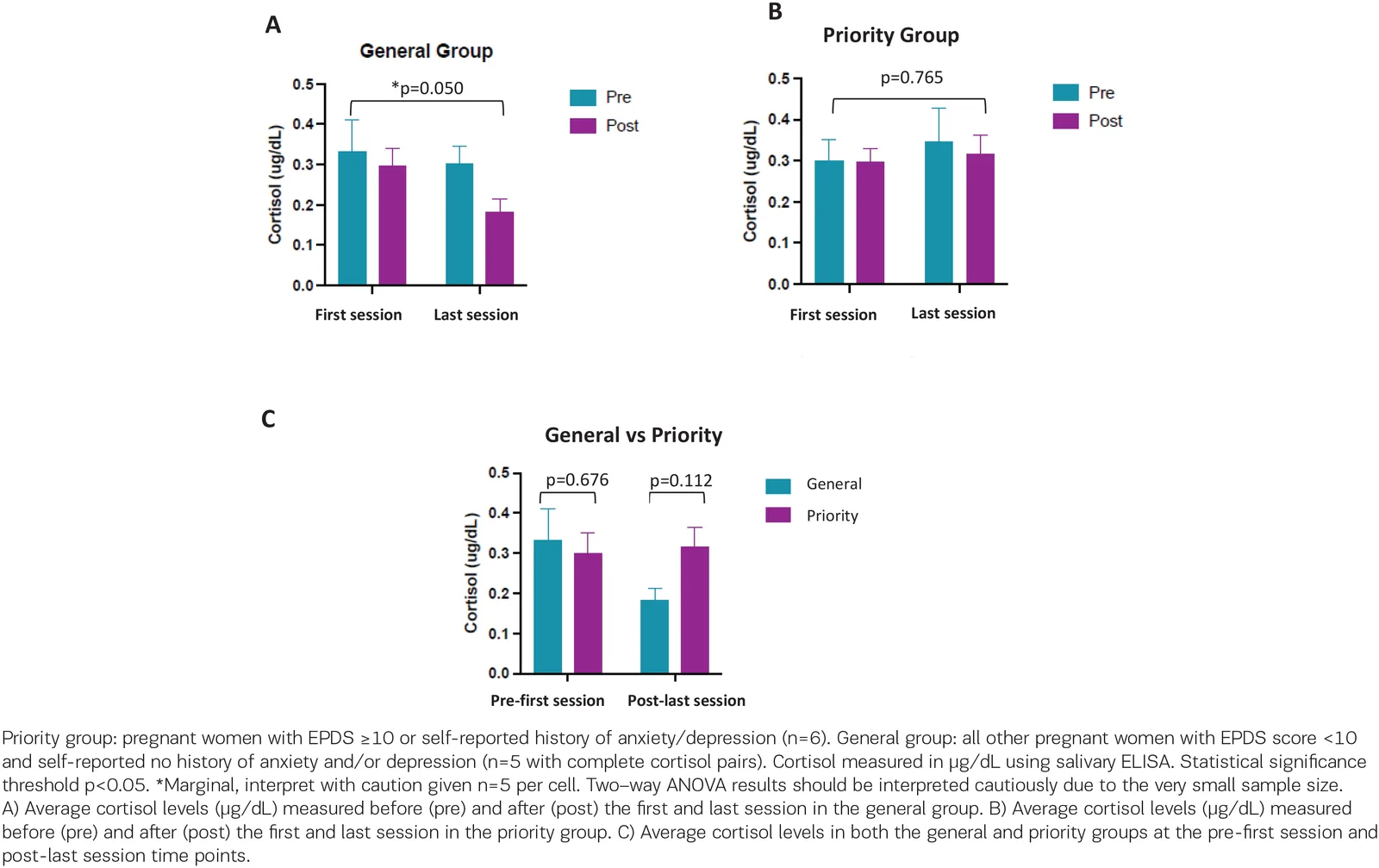
Mean cortisol levels (μg/dL) by group, session, and time point, antenatal clinic, NSW Australia, April 2024–January 2025 (N=13)

Figure 2 shows cortisol levels (μg/dL) for the general and priority groups across sessions and pre–post time points. Two-way ANOVA revealed no significant main effects for group, session, or time (all p>0.05). A marginal difference in cortisol levels between the pre-first session and post-last session time points was observed in the general group (p=0.050, F(1,8)=5.30, d=1.15). Pearson correlation analysis showed no significant association between cortisol levels (μg/dL) and DASS-21 stress subscale scores (r=0.14, p=0.546).

*Evaluation of the intervention*

**Figure 3:**
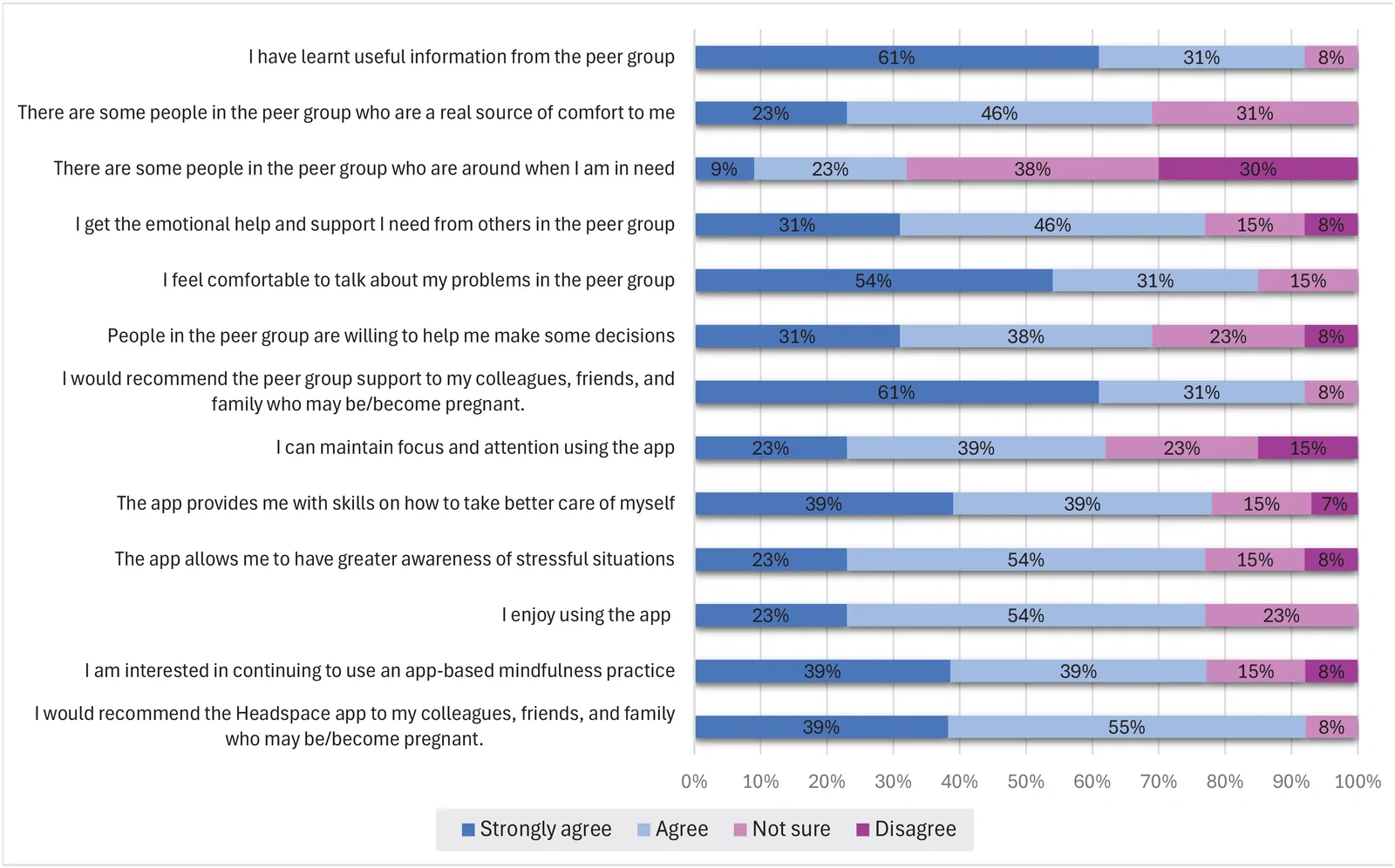
Feedback by participants on the peer support combined with a mindfulness app intervention, antenatal clinic, NSW, Australia, April 2024–January 2025 (N=13)

Intervention feedback is shown in Figure 3. Most participants agreed or strongly agreed that they learned useful information from the peer group (92%) and felt comfortable discussing problems (85%). Many reported receiving emotional help and comfort (78%), and 92% would recommend the peer group to others. Regarding the application, most participants felt it provided useful self-care skills (78%) and increased awareness of stress (77%); 78% expressed interest in continued use and 94% would recommend it to others. However, 46% reported lower confidence in maintaining focus when using the application, and 23% expressed mixed views about its benefits during stressful situations.

### Qualitative findings

Most comments contained some supportive element of social support theory, with emotional support emerging most prominently in the priority group and informational support in the general group, whereas instrumental support occurred infrequently. Due to overlap between the two groups, findings are presented in an integrated manner and are reported in order of frequency, from most to least common.

### Emotional support

More than half of participants reported receiving emotional support through the peer group and mindfulness practice, characterized by feelings of belonging, understanding, and shared experiences. Connecting with women facing similar challenges was central to this support, as one participant noted:

*‘Talking with other women who are going through the same thing.’* (P1)

These interactions fostered emotional connection and validation, with another participant describing it as:

*‘Fantastic to talk to people experiencing similar things and feel heard and be supported.’* (P3)

Participants also reported increased connection and empowerment, with one stating:

*‘The level of support, assistance, advice, discussion, and encouragement is unparalleled elsewhere.’* (P10)

and another describing the experience as:

*‘Connection, sharing knowledge, sharing emotions. Feeling like you are not alone and amplifying each other’s energy.’* (P13)

A safe and non-judgmental space was highlighted as a unique form of emotional support in the intervention. Participants described how this environment allowed them to share their experiences and emotions freely, as one participant stated:

*‘Being present with other pregnant women and sharing experiences and emotions in a safe, non-judgmental space.’* (P6)

while another noted:

*‘A regular meeting time to focus on mental health related to pregnancy in a safe, supportive space.’* (P4)

Participants highlighted that combining peer support with the mindfulness application provided the greatest emotional benefits, noting:

*‘Peer support and feeling calm.’* (P7)

and that:

*‘The mindfulness application allows room for calmness and reflection in an environment that can be quite emotional.’* (P12)

One participant emphasized the value of both components together, stating:

*‘You need both elements for the most enhanced experiences.’* (P11)

However, while some participants valued mindfulness practice, one felt the peer support group was more beneficial, noting:

*‘I do not think the application benefited me more than the peer support group.’* (P8)

### Informational support

Informational support was expressed by participants in terms of the value of gaining strategies and resources through peer exchange.

Participants highlighted the group’s role in facilitating informational support through sharing knowledge and resources. One participant described:

*‘The useful and valuable aspects are the immense valuable knowledge and experience gained over the session that cannot be measured elsewhere.’* (P11)

while others emphasized exchanging practical information, such as:

*‘Receiving information and resources about what others are using and find helpful.’* (P9)

and:

*‘It is a good support group to help and share information.’* (P13)

Some participants noted they could pass this knowledge on to friends, with one stating:

*‘I have friends who are also pregnant and able to share and set information around pregnancy.’* (P12)

The mindfulness application was also viewed as a useful source of practical skills, including:

*‘Practicing the breathing technique and learning how to be present.’* (P10)

with another participant describing it as:

*‘The mindfulness application is a great resource.’* (P7)

### Appraisal support

Appraisal support emerged in comments from participants in the priority pregnancy group, who reported that regular group sessions helped maintain accountability to both the intervention and personal goals. One participant highlighted the value of *‘regular check-ins to determine and assess goals’* (P3), while another noted that practicing mindfulness in a group fostered shared responsibility for progress:

*‘Great to intentionally focus on mindfulness and be held accountable for successes.’* (P2)

### Instrumental support

Instrumental support was the least frequently reported form of social support. Direct practical assistance or material and financial aid were not identified in participant comments; however, participants expressed willingness to support one another within the peer group. One participant expressed that:

*‘I would be more than happy to extend the duration of the pregnancy peer support group.’* (P11), noting that women need as much support and help as possible.

## DISCUSSION

### The feasibility and acceptability of the intervention

This study aimed to evaluate the feasibility of a novel combined peer support and mindfulness application intervention for pregnant women. Key feasibility findings included: low recruitment rates (particularly in pregnant women at risk of anxiety and/or depression), 100% retention, high acceptability based on participant satisfaction, and a statistically significant between-group difference in post-intervention stress (to be interpreted with caution). No statistically significant within-group pre-to-post changes were observed for any psychological outcome. However, qualitative findings suggested that combining peer support with mindfulness promoted emotional support, connection, and engagement in practice. Participants valued the program and were willing to recommend it to others.

Recruitment of pregnant women at risk of anxiety and/or depression was limited, highlighting a major feasibility challenge in identifying and enrolling this priority population. Despite multiple recruitment strategies, few women met eligibility criteria or enrolled, constraining sample size and limiting generalizability. Similar recruitment difficulties have been reported in Australian antenatal mindfulness studies targeting women with elevated psychological distress, indicating a persistent challenge in engaging this group^22^. Although posters and social media generated interest, as reported previously^23^, these approaches produced the lowest conversion to enrolment and yielded no participants in the present study. Recruitment via midwives resulted in the highest enrolment among women at higher risk, while direct researcher recruitment was more effective for the general pregnant population.

Despite recruitment challenges, retention was 100%, indicating strong acceptability among both women at higher risk of anxiety and depression and those from the general pregnant population. Although no prior studies have examined combined peer support and mindfulness interventions, this finding aligns with evidence showing peer support adherence rates above 80%^6^ and average attrition of approximately 22.5% for mindfulness-based interventions alone^24^. The integration of peer support with mindfulness is consistent with the high retention observed, though causal attribution is not possible given the non-randomized design. Evaluation surveys indicated overall satisfaction with the user experience, and qualitative feedback highlighted appreciation for the flexible format and the combined peer support–mindfulness approach. Indeed, there is evidence that antenatal peer support can help alleviate feelings of isolation and foster a sense of empowerment among pregnant women^25^. Although one participant preferred peer support over mindfulness practice, they reported they would still recommend both components to pregnant friends.

### Preliminary outcomes (exploratory)

This study was designed to evaluate the intervention’s practicality and feasibility rather than to identify definitive outcome changes; the outcome data presented must, therefore, be interpreted with caution. Nevertheless, these findings suggest some evidence of between-group differences over time among women who participated in the intervention. Given the small sample size and non-randomized design, these between-group findings should be interpreted with caution and considered hypothesis-generating rather than confirmatory.

Post-intervention stress scores were lower in the general group than in the priority group. However, the priority group had higher baseline stress levels, although this difference was not statistically significant. The post-intervention difference should therefore be interpreted cautiously, as it may reflect the baseline imbalance rather than a true difference between the groups. Nevertheless, the observed pattern is consistent with prior evidence suggesting mindfulness-based interventions may show differential effects across risk strata and may require adaptation for women with more complex comorbidities^26^. Notably, cortisol levels did not correlate with self-reported stress in either group post-intervention, consistent with prior findings that mindfulness may reduce perceived prenatal stress without significantly altering cortisol-based measures, potentially due to poor adherence to sampling protocols^27^. Given the small sample size and exploratory nature of the analysis, the marginal reduction in cortisol observed in the general group between the first and last sessions should be interpreted cautiously.

No statistically significant within-group changes in anxiety or depression were observed in either the priority or general group. Previous peer support research has reported significant reductions in depression and anxiety, particularly when interventions included postnatal involvement and peer volunteers with lived experience of postpartum depression^28^. Meta-analyses suggest that while peer support during pregnancy may be beneficial, effects are more consistently observed postpartum^6^, possibly due to the immediate challenges after birth (e.g. infant care, breastfeeding, sleep deprivation) that intensify stress and create a critical window for support^29^. Therefore, while symptom reduction may be limited during pregnancy, improvements in loneliness, connectedness and overall self-efficacy remain valuable outcomes, warranting further exploration in future research.

Although no statistically significant pre–post differences were observed, mindfulness outcomes were considered encouraging. Previous studies have shown that longer duration interventions can significantly enhance mindfulness during pregnancy; for example, an 8-week mobile-delivered mindfulness-based intervention improved mindfulness in a randomized controlled trial of 178 participants^30^, and a 6-week application-based mindfulness program increased mindfulness among women with moderate depressive symptoms in a smaller pre–post study^31^. These suggest that a longer intervention period and larger samples are needed to confirm and further explore the effects of combining mindfulness practice with peer support.

Although perceived social support scores did not significantly increase from pre- to post-intervention, the qualitative findings provided valuable insights into participants’ experiences. Responses to open-ended post-intervention questions suggested that the intervention was perceived as beneficial in several ways consistent with social support theory. Emotional support appears to be a key element of the positive participant experiences reported, particularly among women in the priority group, consistent with findings from previous peer support interventions^32^. To our knowledge, however, no previous intervention has used this specific combination of peer support and a mindfulness application. Low emotional support has been identified as significantly associated with antenatal depression in Australian women^33^, with its absence linked to increased feelings of loneliness^34^. Emotional support was particularly critical during the COVID-19 pandemic when many women experienced heightened isolation and loneliness^35^. In some cases, this support was limited when spouses or families were distant or unable to assist directly, creating additional challenges^36^.

Participants’ perceptions of the intervention suggest that combining peer support and mindfulness supported both interpersonal connection and individual coping, offering a possible explanation for the intervention’s acceptability and retention. This aligns with existing evidence that group-based mindfulness can foster connectedness and a supportive environment^26^. Peer support similarly offers emotional safety and validation often lacking in other contexts^32^. Consistent with participants’ accounts of valuing being heard and feeling able to express emotions they might otherwise suppress. Although peer support and mindfulness have typically been examined separately, these preliminary findings suggest possible added value in combining both, though this warrants confirmation in future controlled studies.

### Limitations

Several methodological limitations should be acknowledged. The small sample size limited statistical power, and the absence of a control group precludes definitive attribution of observed changes to the intervention. The non-randomized design without a control group means that observed changes in outcomes cannot be attributed to the intervention. Confounding factors such as natural symptom variation cannot be excluded. The use of a convenience sampling strategy also introduces selection bias, as participants who volunteered to take part may differ systematically from non-participants in motivation. Generalizability is also limited by the self-selected sample, single-site setting at one public hospital in New South Wales, and the predominantly employed, first-time mother participants, who may not represent the broader antenatal population or other healthcare and cultural contexts. In addition, the expansion of inclusion criteria may have created a heterogeneous sample with differing baseline risk profiles. The multiple comparisons performed without adjustment increase the risk of Type I error; therefore, the significant between-group difference in stress should be considered hypothesis-generating rather than confirmatory. Self-report bias may also have affected psychological outcome measures, as participants may have responded in socially desirable ways or had difficulty recalling their psychological states. The salivary cortisol analysis should be considered exploratory because the very small cell sizes made it difficult to verify ANOVA assumptions. Finally, without follow-up assessment, it is unknown whether any observed changes or participant-reported benefits were maintained beyond the four-week intervention.

## CONCLUSIONS

This novel intervention, combining peer support with a mindfulness application, was evaluated for feasibility in relation to pregnant women’s mental health, mindfulness practice, and perceived social support. Recruitment challenges limited statistical power, indicating the need for improved recruitment strategies in future trials. Some benefits were observed in emotional support; however, negative mental health outcomes, including affect, anxiety, depression, and stress, did not demonstrate significant improvement. Future research should employ adequately powered randomized controlled trial designs with control conditions, longer intervention durations, including possible extension into the postpartum period, and follow-up assessments to determine whether benefits are maintained over time and can be attributed to the combined intervention.

## Supplementary Material



## Data Availability

The data supporting this research can be found in the Supplementary file.
